# Photodynamic diagnosis of breast tumours after oral application of aminolevulinic acid

**DOI:** 10.1054/bjoc.2000.1532

**Published:** 2001-01-01

**Authors:** D P Ladner, R A Steiner, J Allemann, U Haller, H Walt

**Affiliations:** Women's Hospital Fontana, Luerlibadstrasse 118, Chur, 7000, Switzerland; Research Division of Gynaecology, Department of Ob/Gyn, University Hospital Zurich, Frauenklinikstrasse 10, Zurich, 8091, Switzerland; Cantonal Hospital Chur, Department of Pathology, Loestrasse 170, Chur, 7000, Switzerland

**Keywords:** aminolevulinic acid, photosensitizer, photodynamic diagnosis, photodynamic therapy, breast tumours, axillary lymph nodes

## Abstract

Photodynamic diagnosis is of increasing interest for diagnosis in oncology. It is based on a more intense incorporation of a fluorescent dye in tumours compared to normal tissue. As a feasibility study we investigated the effectiveness of oral application of 5-aminolevulinic acid for photodynamic diagnosis of human primary mammary tumours. The study included 16 patients with palpable breast tumours. Aminolevulinic acid was administered at a concentration of 40 mg kg^−1^bodyweight 150–420 min prior to tumourectomy. Intraoperatively blue light (405 nm) was applied to the operation site. Sections of the excised tumour and some lymph nodes were prepared and analysed with a fluorescent microscope. All primary mammary tumour tissues showed significantly higher fluorescence intensity than surrounding normal mammary tissue. Fluorescence of the mammary tumours could also be discriminated macroscopically and intraoperatively. Fluorescence intensity in nonmetastatic lymph node tissue was higher in 2 out of 3 patients than in primary tumour tissue. By photodynamic diagnosis using aminolevulinic acid we were able to reliably distinguish primary mammary tumours from normal mammary tissue microscopically and macroscopically in all our patients. We suggest that photodynamic diagnosis with aminolevulinic acid for breast tumours should be further investigated and developed for intraoperative use and may well be a simple tool for better intraoperative diagnosis and recognition of tumour margins. We hypothesize that lymph node metastasis of breast tumours will not be detectable by this method. © 2001 Cancer Research Campaign http://www.bjcancer.com


British Journal of Cancer (2001) **84**(1), 33–37
© 2001 Cancer Research Campaign
doi: 10.1054 bjoc.2000.1532, available online at http://www.idealibrary.com on IDEAL®

http://www.bjcancer.com

### Photodynamic diagnosis of breast tumours after oral application of aminolevulinic acid

**DP Ladner[1,2], RA Steiner[1], J Allemann[3], U Haller[2] and H Walt[2]**

[1]Women's Hospital Fontana, Luerlibadstrasse 118, 7000 Chur, Switzerland; [2]Research Division of Gynaecology, Department of Ob/Gyn, University Hospital Zurich, Frauenklinikstrasse 10, 8091 Zurich, Switzerland; [3]Cantonal Hospital Chur, Department of Pathology, Loestrasse 170, 7000 Chur, Switzerland

**Summary** Photodynamic diagnosis is of increasing interest for diagnosis in oncology. It is based on a more intense incorporation of a fluorescent dye in tumours compared to normal tissue. As a feasibility study we investigated the effectiveness of oral application of 5-aminolevulinic acid for photodynamic diagnosis of human primary mammary tumours. The study included 16 patients with palpable breast tumours. Aminolevulinic acid was administered at a concentration of 40 mg $kg^{-1}$ bodyweight 150–420 min prior to tumourectomy. Intraoperatively blue light (405 nm) was applied to the operation site. Sections of the excised tumour and some lymph nodes were prepared and analysed with a fluorescent microscope. All primary mammary tumour tissues showed significantly higher fluorescence intensity than surrounding normal mammary tissue. Fluorescence of the mammary tumours could also be discriminated macroscopically and intraoperatively. Fluorescence intensity in nonmetastatic lymph node tissue was higher in 2 out of 3 patients than in primary tumour tissue. By photodynamic diagnosis using aminolevulinic acid we were able to reliably distinguish primary mammary tumours from normal mammary tissue microscopically and macroscopically in all our patients. We suggest that photodynamic diagnosis with aminolevulinic acid for breast tumours should be further investigated and developed for intraoperative use and may well be a simple tool for better intraoperative diagnosis and recognition of tumour margins. We hypothesize that lymph node metastasis of breast tumours will not be detectable by this method.
© 2001 Cancer Research Campaign http://www.bjcancer.com

**Keywords:** aminolevulinic acid; photosensitizer; photodynamic diagnosis; photodynamic therapy; breast tumours; axillary lymph nodes

For the detection of tissue alterations progressing to a malignant tumour, relevant diagnostic instruments and methods are crucial. This especially is true for detection of early and/or small lesions, which are not visible macroscopically. Two decades ago, photodynamic diagnosis (PDD) was introduced; its use is increasingly important in gynaecology (Dougherty and Marcus, 1992). PDD is based on the administration of a photosensitizer (PS) to cells and tissues, the subsequent exposure of the tissue to light of specific wavelength and the detection of a characteristic fluorescence-emission (Henderson and Dougherty, 1992). This is of clinical importance since certain PS preferentially accumulate in tumorous tissue (Kennedy and Pottier, 1994).

Various substances have been studied including porphyrins, synthetic porphyrins and phthalocyanins. Modified porphyrins have systemically been applied for PDD of tumour tissue since the early 1980s, although the fluorescence signal within the tissue was not significant, making a clinical use of this method difficult (Kato et al, 1984). 10 years ago, aminolevulinic acid (ALA) was found effective for both PDD and photodynamic therapy (PDT) (Kennedy et al, 1990; Gibson et al, 1997; Peng et al, 1997). Protoporphyrin IX (PPIX), an endogenously produced porphyrin and a metabolite of the haem synthesis induced by ALA, accumulates in the mitochondria and can serve as a PS; ferrochelatase is the rate-limiting step for the incorporation of iron into PPIX. In some tumours, deaminase-isomerase, the rate-limiting enzyme that converts porphobilinogen into uroporphyrinogen in the haem synthesis pathway is increased and ferrochelatase is reduced, so that PPIX accumulates preferentially in tumour cells (Hua et al, 1995; Heyerdahl et al, 1997; Peng et al, 1997). Photosensitization by ALA-induced PPIX is tissue specific and is primarily enhanced in tissues that cover body surfaces, or line body cavities and exocrine glands such as mammary glands (Kennedy and Pottier, 1994; Marcus et al, 1996; Riesenberg et al, 1996).

Clinical studies with PDD and PDT using ALA were performed for the treatment of neural tumours (Obwegeser et al, 1998), lesions of the oral cavity (Leuing et al, 1996), endoscopical detection of bronchial lesions (Campbell et al, 1996) and detection of carcinoma in situ of the bladder during cystoscopy (Kriegmair et al, 1994). The latter reached a sensitivity of 93%. Applications have been developed for detection of alterations in gynaecological tissues including the endometrium (Gannon et al, 1995; Steiner et al, 1996), the vulva (Fehr et al, 1996) and skin metastasis of the breast (Khan et al, 1993).

Although high levels of PPIX were found in mammary tumour tissue in vitro (Navone et al, 1990), in mice (Peng et al, 1992) and in women (Kennedy and Pottier, 1992), no clinical study has been reported using ALA-PDD for primary mammary tumours.

In the present report, as a feasibility study, we investigated the effectiveness of oral application of ALA for PDD in patients with primary mammary tumours. We monitored the fluorescence intensity (FI) of mammary tissue by fluorescence microscopy and compared tumorous mammary tissue with normal mammary tissue and lymph node tissue. We also investigated intraoperative application of PDD with ALA for primary mammary tumours.

*Received 27 April 2000*
*Revised 10 August 2000*
*Accepted 13 September 2000*

*Correspondence to:* H Walt

### PATIENTS, MATERIALS AND METHODS

The study was performed at the Women's Hospital Fontana, Chur, Switzerland. After the approval of the ethical committee in Chur,

Graubunden, Switzerland, on October 28, 1998, patients with a palpable breast tumour with written informed consent were enrolled in this study. Patients with the following clinical findings were excluded: porphyria, high levels of liver enzymes, insufficient function of the liver or the kidney and pregnancy.

### Aminolevulinic acid

δ-Aminolevulinic acid from ASAT AG, Zug, Switzerland was used. 16 patients received 40 mg $kg^{-1}$ bodyweight ALA, which was freshly dissolved in 50 cl of tap water (pH 2–4) and was applied 3–6 hours prior to surgery.

### Protection of patients

Patients were given Ondansetron (Zofran®) 8 mg together with ALA. A sun blocker Spirig® (Spiring Pharma AG, Egerkingen, Switzerland) was applied to face and neck then covered with a micropigment cream, which has shown good results in light protection during photosensitization with ALA (unpublished results). In addition patients were protected from direct light for 24 hours after application of ALA.

### Tumours

The excised palpable tumours, as well as the lymph nodes, were protected from light in sealed bottles and transported immediately to the pathology department, Cantonal Hospital Chur, Switzerland. Tumours and the lymph nodes were carefully divided into two parts by a surgical blade and were protected from light. One part underwent the regular pathological diagnostic procedures. The other part was stored and frozen in a light protected container at –80˚C. In a dark chamber 10 cryosections, 10 μm thick were prepared from every tumour in the dark. The excised tissue was cut in a way that there was normal and altered tissue on the same slide. The sections were wrapped in aluminium foil, sealed in boxes protecting against drying out and photobleaching, then stored at –80˚C. These slides were transported on dry ice to the Research Division of Gynaecology, University Hospital, Zurich. One section was stained with haematoxylin & eosin for conventional histological information and orientation of unstained sections. Unstained sections were analysed by fluorescence microscopy. From every tumour and the corresponding normal tissue, 3 sections were analysed. On every section, 3 pictures (512 $pixel^2$) of the tumour were compared with the FI of 3 pictures (512 $pixel^2$) taken from normal mammary tissue. The analysed sections were additionally stained with haematoxylin & eosin and histological information was compared with the digitized FI.

### PDD during surgery

During a second step, intraoperative PDD was performed. Tumours were cut into half and then illuminated macroscopically using a Minolta camera on a stativ (Minolta Dynay 500si with an AF 100 mm objective 1:2.8 (32)) with a diaphragm opening of 5.6. After tumourectomy, the operation field was illuminated; fluorescence was detected with a light source (Intralux® mdr100, Volpi AG, Schlieren, Switzerland). A Xenon lamp 100 W, spectrum 400–780 nm with a liquid light conductor of 15 cm length, an active fibre diameter of 8 mm and a filter of 430 nm S-10 410 (transmission band 45%, bandwidth 10 nm) served as a light source.

### Fluorescence microscopy

Fluorescence imaging was performed with a CCD camera system (Photometrics, Tucson, AZ, USA) with a Kodak KAF 1600 chip, coupled to a Leitz DMRB/E fluorescence microscope (Leica, Glattbrugg, Switzerland). Light from a 50 mW HBO mercury lamp was filtered through a 405–420 nm broad-bandpass filter to provide excitation light. A dichroic mirror reflected the excitation light onto the sample and transmitted the fluorescence emission through a 635–655 nm broad-bandpass filter onto the detector. Instrument control, image acquisition, and processing were performed with a Macintosh computer and IPlab® software (Scanalytics, Fairfax, VA, USA). Tissue images were acquired with an integration time of 1 s. The FI of the tumour pictures and normal mammary tissue pictures of every slide were each averaged and the tumour/normal mammary tissue ratio was used for comparison. FI was expressed as counts per pixel (cpp, arbitrary units). All optical settings of the microscope were kept constant for an entire series of image acquisition.

### Statistical analysis

Comparison of the effectiveness of ALA-based PDD was made between the group after ALA application and the control, and between ALA-treated tumours and normal tissue by applying the nonparametric Mann-Whitney test and Student's *t*-test. Stat View® Software (SAS Institute Inc., Cary, NC, USA) was used for statistical analysis and $P < 0.05$ was considered to be a statistically significant difference.

### RESULTS

### Fluorescence microscopy

*Fluorescence intensity of mammary tumours compared with surrounding normal mammary tissue*

Sections from 13 out of 16 mammary tumours were examined, after pre-surgical oral application of 40 mg ALA $kg^{-1}$ bodyweight.

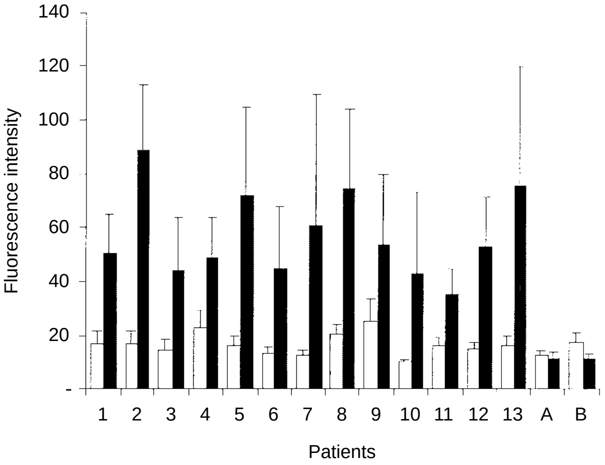

**Figure 1** Fluorescence intensity (FI) of tumour tissue versus normal surrounding tissue. Columns represent means of data ± SD. Dark columns: FI of tumour-tissue. White columns: FI of normal surrounding tissue. (1–13) Tissue of 13 patients after oral intake of 40 mg ALA $kg^{-1}$ bodyweight. (A) Auto-fluorescence of a fibroadenoma versus surrounding normal tissue, (B) Autofluorescence of a carcinoma versus surrounding normal tissue.

© 2001 Cancer Research Campaign

**Table 1** List of patients

| Patients[a] | Tumour[b] | Normal tissue[c] | Ratio[d] | *t*-test[e] | FA/CA[e] | Time[f] |
|---|---|---|---|---|---|---|
| 1 | 50.62 ± 14.2 | 17.0 ± 4.49 | 3.0 | 0.0010 | FA | 420 |
| 2 | 88.64 ± 24.88 | 17.03 ± 4.2 | 5.2 | 0.0001 | CA | 205 |
| 3 | 44.13 ± 19.42 | 14.73 ± 4.06 | 3.0 | 0.0002 | CA | 150 |
| 4 | 48.92 ± 14.52 | 22.51 ± 6.92 | 2.2 | 0.0024 | FA | 230 |
| 5 | 72.19 ± 32.84 | 15.87 ± 4.26 | 4.6 | 0.0008 | CA | 180 |
| 6 | 44.54 ± 23.57 | 13.14 ± 2.41 | 3.4 | 0.0120 | FA | 250 |
| 7 | 61.08 ± 48.31 | 12.33 ± 2.12 | 5.0 | 0.0004 | FA | 310 |
| 8 | 74.8 ± 29.25 | 20.38 ± 3.42 | 3.7 | 0.0039 | FA | 200 |
| 9 | 53.26 ± 26.49 | 25.45 ± 7.75 | 2.1 | 0.0112 | CA | 240 |
| 10 | 42.88 ± 30.38 | 10.06 ± 1.04 | 4.3 | 0.0002 | FA | 240 |
| 11 | 35.09 ± 9.79 | 16.42 ± 2.88 | 2.1 | 0.0002 | CA | 270 |
| 12 | 52.73 ± 18.77 | 15.32 ± 1.88 | 3.4 | 0.0004 | CA | 230 |
| 13 | 75.4 ± 45.1 | 16.08 ± 3.6 | 4.7 | 0.0014 | CA | 310 |
| A | 11.68 ± 2.0 | 12.76 ± 1.45 | 0.9 | 0.2080 | FA | 0 |
| B | 11.43 ± 1.7 | 17.3 ± 3.54 | 0.7 | 0.0010 | CA | 0 |

[a]Patients 1–13 were photosensitized prior to surgery with 40 mg ALA $kg^{-1}$ bodyweight. Patients A and B served as controls: no application of ALA. bMean of FI ± SD of benign or malignant tumour tissue. [c]Mean of FI ± SD of surrounding normal mammary tissue. [d]Ratio of means of FI tumour tissue/normal mammary tissue. [e]Student's *t*-test: Difference between FI of tumour tissue versus normal surrounding mammary tissue. [e]FA: fibroadenoma, CA: carcinoma. [f]Time interval between oral intake of ALA and tumourectomy.

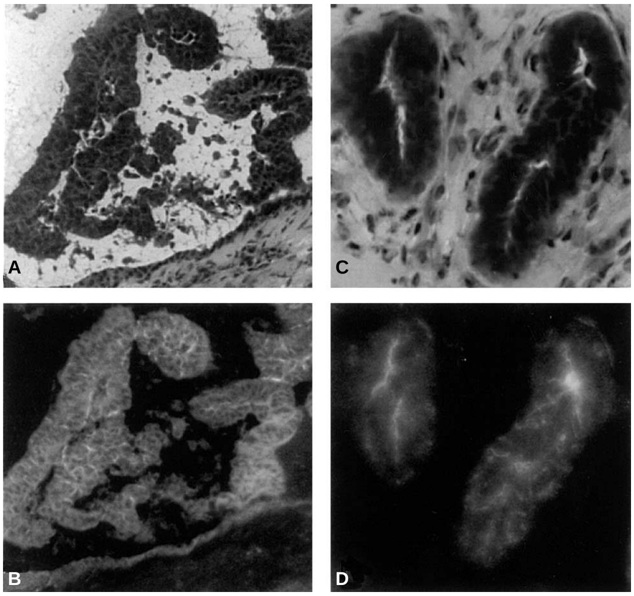

**Figure 2** Fluorescence microscopy (20 ×) of tumour versus normal mammary tissue after oral photosensitization with ALA (**B**, **D**) with corresponding pictures after staining with haematoxylin & eosin (**A**, **C**). (**B**) Fluorescence picture of fibroadenoma, (**D**) Fluorescence picture of normal mammary tissue.

Two collected tumours did not show any normal tissue and comparative fluorescence assessment could therefore not be performed. Tissue of one patient who received ALA was held back because of change of surgical procedure. The histological report of the 13 mammary tumours, each from a different patient, revealed six grade 2 carcinomas (4 cases: ductal invasive carcinoma, 2 cases: lobular carcinoma), one grade 1 carcinoma (mucinous carcinoma) and six fibroadenomas. Every tumour showed a highly significant difference in fluorescence intensity compared to the surrounding normal mammary tissue (Table 1). FI from tumour tissue was 2.2–5.2 fold higher than normal mammary tissue. FI of tumour tissue ranged from 35.1–75.4, normal mammary tissue ranged from 10.1–25.5. We generated a ratio between the tumours and the normal surrounding mammary tissue in order to compare the tumours with each other. We have chosen this approach because the absolute intensity of fluorescence varied from patient to patient. The mean ratio ± SD was 3.59 ± 1.09. No significantly different ratio was found between benign and malignant tumours. Autofluorescence of the tumour tissue, considered as the background fluorescence of the tissue, without additional exposure to ALA was measured in one patient with fibroadenoma and in one with carcinoma. There was no increased FI measured in tissue of controls (Figures 1, 2).

### *Fluorescence intensity of axillary lymph nodes after application of ALA*

We examined 8 lymph nodes from 3 patients who had axillary lymphonodectomy after tumourectomy of the breast during the same surgical session. The macroscopic appearance of the lymph nodes was normal. The histological report on all the lymph nodes was negative for malignancy. We hypothesized that in order to see tumour metastasis fluorescing in the lymph node, the lymph nodes tissue itself must have a lower fluorescence than the tumour tissue. The mean ±SD FI of the lymph node tissue was as follows: photosensitized lymph nodes: 50.57 ± 35.8, autofluorescence of lymph nodes 14.53 ± 3.7. The difference was highly significant ($P$ = 0.0002). The mean ±SD of FI in patient A (28.4 ± 17.1) was considerably lower than in patient B (61.7 ± 30.8) and patient C (45.9 ± 25.2). However, when comparing FI of the nonmetastatic lymph node tissue with FI of the primary breast tumour of the same patient, only patient A revealed significantly higher FI in the mammary tumour tissue than in the lymph node tissue ($P < 0.001$). Patients B and C showed no significant difference (B: $P = 0.44$, C: $P = 0.55$).

### *Comparison of fluorescence intensity of tumours with time between ALA application and tumour excision*

Tumours were excised between 150–420 minutes after oral application of 40 mg ALA $kg^{-1}$ bodyweight. 11 out of 13 tumours (85%) were excised within 180–320 minutes post photosensitization. There was no correlation between excision latency and FI or ratio of FI (Table 1).

*© 2001 Cancer Research Campaign* 

### Intraoperative fluorescence of tumours

### *Whole mount fluorescence in tumours*

Tumours were excised, cut into half and exposed to blue light. Intense red fluorescence was shown (Figure 3). Intraoperatively, tumours could also be detected and described after darkening the room and exposure to blue light (not recorded).

### Side effects from oral application of ALA

Oral application of ALA at a concentration of 40 mg $kg^{-1}$ bodyweight had little and short-lived side effects. Prior to this study 5 patients (unpublished data) who were photosensitized with 60 mg ALA $kg^{-1}$ bodyweight developed the following side effects: 3 patients developed a facial erythema in the evening and oedema of the eyelids. They had paranasal swelling with an itchy feeling but recovered completely within 72 hours. One patient had severe nausea 2 hours after intake of ALA and 2 patients had postoperative nausea. Due to these side effects the concentration of ALA was reduced to 40 mg $kg^{-1}$ bodyweight for this study. With this concentration applied to 16 patients, 2 patients developed a facial erythema in the evening, but without itching. Perinasal swelling was a common finding which affected 75% of the patients without subjective distress. All of them recovered within 72 hours completely. There was one patient with severe sunburn that lingered for 5 days resulting from a combination with homeopathic medication (Hypericin extract). The patient had not mentioned the medication prior to surgery. This substance is known for its photosensitizing side effects and we could prove synergistic effect with ALA in vitro (unpublished data).

### DISCUSSION

All mammary tumour samples examined by fluorescence microscopy presented a significantly higher fluorescence intensity than the normal surrounding tissue. Autofluorescence was very low and similar in both normal and tumour tissue of the breast. This corresponds to earlier findings in mice (Peng et al, 1992). In addition, intraoperative fluorescence of tumour tissue after initial operative exposure before and after removal emphasizes the potential of this new tool to discriminate primary mammary tumours from normal tissue intraoperatively. Further characterization of the tumour tissue will rely on the pathologist, since FI between benign and malignant tumours did not show any significant difference. We believe that this method will be of help to determine the extent of tumour involvement and therefore give the surgeon real-time information on whether a sufficient margin has been allowed, resulting in an increased radicality in breast tumour management. This has been proposed previously for mammary tumours after i.v. application of Photofrin (Svanberg et al, 1998). The qualities of ALA however, make a clinical application for PDD more feasible.

Kriegmair et al have used this procedure to detect carcinoma in situ of the human bladder (Kriegmair et al, 1994). The present study only evaluated women with palpable tumours. Because of the presence of a significant difference in fluorescence intensity of altered and normal mammary tissue, we believe that this method may well have the potential to detect small breast tumours or carcinoma in situ. It will therefore be interesting to further pursue this issue with regards to nonpalpable tumours and eventually carcinoma in situ. Furthermore, we consider a combination of PDD with PDT as a possible approach to combine diagnosis with treatment. PS of the most recent generation have their spectrum of absorption at higher wavelength (650–740 nm) enabling deeper penetration into the tissue and thus enhancing the therapeutic effect of the procedure.

We found a large variation in FI among our patients. This is in accord with the literature stating that many factors influence the amount of PPIX accumulated in the tissue. For example, FI increases exponentially over the pH range between 6 and 8 (Krammer and Uberriegler 1996). In addition, metabolism of ALA varies from one patient to another. We therefore have chosen a specific ratio of FI in the tumour and in the surrounding tissue enabling a comparison between all patients.

Various ways of ALA administration have been studied. Topical administration was very efficient and did not show relevant side effects. Targeting the PS to the tumour, especially when it is not located on the surface, however, is a problem for topical application. This can be overcome by endogenous photosensitization with oral or intravenous administration of ALA. It was shown that oral ALA administration takes more time than intravenous application to induce PPIX fluorescence in the various tissues, but shows fewer side effects (Van den Boogert et al, 1985; Loh et al, 1993). Oral application of ALA is thus accompanied by systemic photosensitization, but in contrast to other porphyrins, which stay within the body for many days or weeks, photosensitization by ALA is

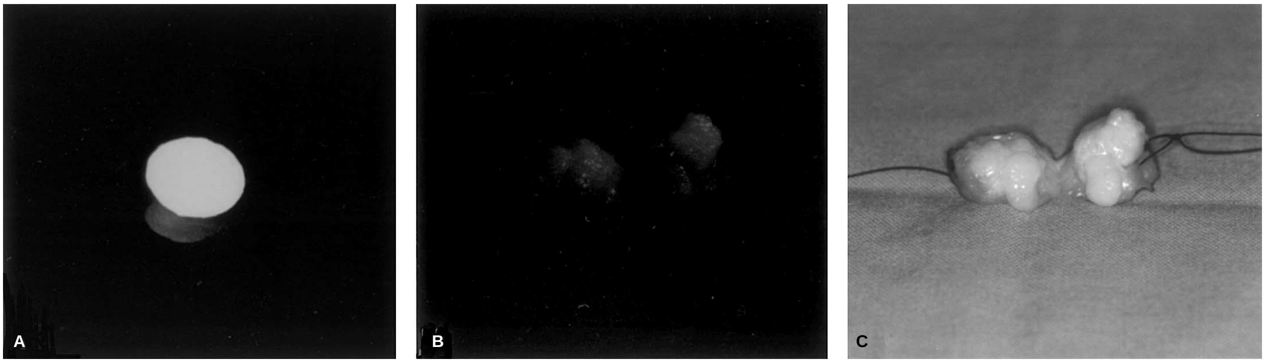

**Figure 3** Photosensitized (**A**) and nonphotosensitized (**B**, **C**) fibroadenoma cut into half. The surface of the photosensitized tumour shows intense fluorescence during exposure to blue light (**A**). Nonphotosensitized fibroadenoma (**B**, **C**) is barely visible during exposure to blue light (**B**) compared to exposure with white light (**C**).

© 2001 Cancer Research Campaign limited to 24 hours after intake. Dose related side effects have been examined with the conclusion that doses of 60 mg $kg^{-1}$ bodyweight are acceptable but more likely to produce side effects such as nausea and phototoxic reaction of the light exposed skin, than 40 mg $kg^{-1}$ (Webber et al, 1997). On the other hand, the higher dose is expected to produce a higher FI. The chosen dosage of 40 mg $kg^{-1}$ bodyweight was convenient to the patients in almost all cases. However, paranasal oedema was a common finding that had not been reported before. Such side effects after oral intake of ALA will need to be studied in greater detail.

As for lymphatic tissue, it has been shown that PPIX accumulates preferentially in active lymphatic tissue (Eleouet et al, 1997) such as lymphomas. Nonmetastatic lymph nodes in 2 out of 3 patients showed high FI. In fact, the FI of the nonmetastatic lymph node tissue was as high as the FI in the primary mammary tumours of the same patient. We therefore assume that metastasis of the breast tumour could not be detected in the lymph nodes by our means, although the number of lymph nodes examined does not allow a final conclusion.

Based on this feasibility study, we suggest that PDD with ALA for breast tumours should be further investigated and developed for intraoperative use and may be a simple tool for intraoperative diagnosis and safe recognition of tumour margins.

### ACKNOWLEDGEMENTS

The authors acknowledge A Kurmanaviciene for fluorescence microscopy, A Mueller, ETH Zurich, Switzerland, for statistical advice, C Heinz, Cantonal Hospital Chur, Switzerland, Department of Pathology for histological preparations and F Schleiss, P Fehr, I Strnad, Woman's Hospital Fontana, Chur, Switzerland, for participation and support in clinical trial.

This study was supported by a grant from the Swiss Med Tech Initiative programme, dedicated to the improvement of ALA substances (Grant No 551,3804), by ASAT AG, Zug and by Volpi AG. Schlieren, Switzerland.

### REFERENCES

Campbell DL, Gudgin-Dickson EF, Forkert PG et al (1996) Detection of early stages of carcinogenesis in adenoma of murin lung by 5-aminolevulinic acid-induced protoporphyrin IX fluorescence. *Photochem Photobiol* **64**(4): 676–682

Dougherty TJ and Marcus SL (1992) Photodynamic therapy. *Eur J Cancer* **28A** (10): 1734–1742

Eleouet S, Carre J, Vonarx V et al (1997) Delta-aminolevulinic acid-induced fluorescence in normal human lymphocytes. *J Photochem Photobiol B* **41**: 22–29

Fehr MK, Chapman CF, Krasieva T et al (1996) Selective photosensitizer distribution in vulvar condyloma acuminatum after topical application of 5-aminolevulinic acid. *Am J Obstet Gynecol* **174**(3): 951–957

Gannon MJ, Johnson N, Roberts DJH et al (1995) Photosensitization of the endometrium with topical 5-aminolevulinic acid. *Am J Obstet Gynecol* **173**(6): 1826–1828

Gibson SL, Havens JJ, Foster TH and Hilf R (1997) Time-dependent intracellular accumulation of delta-aminolevulinic acid, induction of porphyrin synthesis and subsequent phototoxity. *Photochem Photobiol* **65**(3): 416–421

Henderson BW and Dougherty TJ (1992) How does photodynamic therapy work? Review. *Photochem Photobiol* **55**: 145–157

Heyerdahl H, Wang I, Liu DL et al (1997) Pharmacokinetics studies on 5-aminolevulinic acid-induced protoprophyrin IX accumulation in tumors and normal tissue. *Canc Lett* **112**: 225–231

Hua Z, Gibson SL, Foster TH and Hilf R (1995) Effectiveness of delta-aminolevulinic acid-induced protoporphyrin as a photosensitizer for photodynamic therapy in vivo. *Cancer Research* **55**: 1723–1731

Kato H, Okunaka T and Shimatani H (1984). Clinical measurement of tumor fluorescence using a new diagnostic system with hematoporphyrin derivative, laser photoradiation, and a spectroscope. *Lasers Surg Med* **4**(1): 49–58

Kennedy JC and Pottier RH (1992) Endogenous protoporphyrin IX, a clinically useful photosensitizer for photodynamic therapy. *J Photochem Phtobiol B* **14**: 275–292

Kennedy JC and Pottier RH (1994) Using delta-aminolevulinc acid in cancer therapy. *Porphyric pesticides*, Chapter 21

Kennedy JC. Pottier RH and Pross DC (1990) Photodynamic therapy with endogenous protoporphyrin IX: basic principles and present clinical experience. *J Photochem Photobiol B* **6**(1–2): 143–148

Khan SA, Dougherty TJ and Mang TS (1993) An evaluation of photodynamic therapy in the management of cutaneous metastases of breast cancer. *Eur J Cancer* **29A**(12): 1686–1690

Krammer B, Uberriegler K (1996) In-vitro investigation of ALA-induced protoporphyrin IX. *J Photochem Photobiol B* **36**: 121–126

Kriegmair M, Baumgartner R, Knuechel R et al (1994) Fluorescence photodetection of neoplastic urothelial lesions following intravesical instillation of 5-aminolevulinic acid. *Urology* **44**(6): 836–41

Leuing A, Rick K, Stepp H et al (1996) Fluorescence imaging and spectroscopy of 5-aminolevulinic acid induced protoporphrin IX for the detection of neoplastic lesions in the oral cavity. *Am J Surg* **172**: 674–677

Loh CS, MacRobert AJ, Bedwell J et al (1993) Oral versus intravenous administration of 5-aminolaevulinic acid for photodynamic therapy. *Br J Cancer* **68**(1): 41–51

Marcus SL, Sobel RS, Golub AL et al (1996) Photodynamic therapy (PDT) and photodiagnosis (PD) using endogenous photosensitization induced by 5-aminolevulinic acid (ALA): current clinical and development status. *J Clin Laser Med Surg* **14**(2): 59–66

Navone NM, Polo CF, Frisardi AL, et al (1990) Heme biosynthesis in human breast cancer-mimetic "in vitro" studies and some heme enzymic acitivty levels. *Int J Biochem* **22**(12): 1407–1411

Obwegeser A, Jakober R and Kostron H (1998) Uptake and kinetics of 14C-labelled meta-tetrahydroxyphenylchlorin and 5-aminolaevulinic acid in the C6 rat glioma model. *Br J Cancer* **78**(6): 733–738

Peng Q, Moan J, Warloe T et al (1992) Distribution and photosensitizing efficiency of porphyrins induced by application of exogenous 5-aminolevulinic acid in mice bearing mammary carcinoma. *Int J Cancer* **52**(3): 433–443

Peng Q, Warloe T, Berg K et al (1997) 5-Aminolevulinic acid-based photodynamic therapy. *Cancer* **79**(12): 2282–2308

Riesenberg R, Fuchs C and Kriegmair M (1996) Photodynamic effects of 5-aminolevulinic acid-induced porphyrin on human bladder carcinoma cells in vitro. *Eur J Cancer* **32A**(2): 328–334

Steiner RA, Tadir Y, Tromberg BJ et al (1996) Photosensitization of the rat endometrium following 5-aminolevulinic acid induced photodynamic therapy. *Lasers Surg Med* **18**(3): 301–308

Svanberg K, Wang I, Colleen S et al (1998) Clinical multi-colour fluorescence imaging of malignant tumours – initial experience. *Acta Radiol* **39**: 2–9

Van den Boogert J, Van Hillegersberg R, De Rooij FW et al (1985) 5-Aminolaevulinic acid-induced protoporphyrin IX accumulation in tissues: pharmacokinetics after oral or intravenous administration. *J Photochem Photobiol B* **44**(1): 29–38

Webber J, Kessel D and Fromm D (1997) Side effects and photosensitization of human tissues after aminolevulinic acid. *J Surg Res* **68**(1): 31–37

© 2001 Cancer Research Campaign
